# Emergence of IMP-8-Producing *Comamonas thiooxydans* Causing Urinary Tract Infection in China

**DOI:** 10.3389/fmicb.2021.585716

**Published:** 2021-03-15

**Authors:** Xiaobing Guo, Qian Wang, Hao Xu, Xiaohong He, Lihua Guo, Shuxiu Liu, Peipei Wen, Jianjun Gou

**Affiliations:** ^1^Department of Laboratory Medicine, The First Affiliated Hospital of Zhengzhou University, Zhengzhou, China; ^2^State Key Laboratory for Diagnosis and Treatment of Infectious Diseases, The First Affiliated Hospital, College of Medicine, Zhejiang University, Hangzhou, China; ^3^Academy of Medical Sciences, Zhengzhou University, Zhengzhou, China; ^4^Department of Clinical Laboratory, Women and Infants Hospital of Zhengzhou, Zhengzhou, China

**Keywords:** *bla*_*IMP*__–8_, antimicrobial resistance, complete genome sequence, comparative genomic analysis, *Comamonas thiooxydans*

## Abstract

The emergence of carbapenem resistance (CR) caused by hydrolytic enzymes called carbapenemases has become a major concern worldwide. So far, CR genes have been widely detected in various bacteria. However, there is no report of CR gene harboring *Comamonas thiooxydans*. We first isolated a strain of an IMP-8-producing *C. thiooxydans* from a patient with urinary tract infection in China. Species identity was determined using MALDI-TOF MS analysis and carbapenemase-encoding genes were detected using PCR. The complete genomic sequence of *C. thiooxydans* was identified using Illumina Novaseq and Oxford Nanopore PromethION. Antimicrobial susceptibility analysis indicated that the *C. thiooxydans* strain ZDHYF418 was susceptible to imipenem, intermediate to meropenem, and was resistant to aztreonam, fluoroquinolones, and aminoglycosides. The *bla*_*IMP–*__8_ gene was chromosomally located, and was part of a Tn402-like class 1 integron characterized by the following structure: *DDE-type integrase/transposase/recombinase-tniB-tniQ-recombinase family protein-aac(6′)-Ib-cr-bla_*IMP–*__8_-intI1*. Phylogenetic analysis demonstrated that the closest relative of ZDHYF418 is *C. thiooxydans* QYY (accession number: CP053920.1). We detected 330 SNP differences between ZDHYF418 and *C. thiooxydans* QYY. Strain QYY was isolated from activated sludge in Jilin province, China in 2015. In summary, we isolated a strain of *C. thiooxydans* that is able to produce IMP-8 and a novel *bla*_*OXA*_. This is the first time that a CR gene has been identified in *C. thiooxydans*. The occurrence of the strain needs to be closely monitored.

## Introduction

The genus *Comamonas* contains species that are Gram-negative, aerobic, non-pigmented, and rod-shaped bacteria that belong to β-Proteobacteria, which are motile through the use of at least one polar tuft of flagella, and has non-fermentative chemoorganotrophic metabolism. These are quite ubiquitous in the environment and have been isolated from soil, termite guts, activated sludge, humans, fresh water, sediments, and garden ponds. *Comamonas* strains have been found to be isolated from various clinical samples, as well as from the hospital environment. However, they are not seen as pathogenic to healthy humans (Willems and De Vos, 2006; Hatayama, 2014; Narayan et al., 2016; Subhash et al., 2016; Kämpfer et al., 2018). Despite the fact that *Comamonas* spp. are considered to be non-pathogenic or rare opportunistic pathogens to human, some *Comamonas* species have been suggested to be involved in many different infections, including *Comamonas testosteroni*, *Comamonas kerstersii*, and so on (Tsui et al., 2011; Zhou et al., 2018). *Comamonas thiooxydans* is a Gram-negative bacterium that belongs to the genus *Comamona*. *Comamonas* is comprised by 23 species with validly published names^1^. *C. thiooxydans* had the ability to oxidize thiosulfate under mixotrophic growth condition (Narayan et al., 2016). *C. thiooxydans* can grow under anoxic conditions, while other species that belong to *Comamonas* are strictly aerobes (Chen et al., 2016). *Comamonas thiooxydans* is most closely related to *Comamonas testosteroni* (Pandey et al., 2009). Of the all 21 *C. thiooxydans* in Genome of NCBI, 14 of them were originally submitted as *Comamonas testosteroni* but have later changed to *C. thiooxydans* due to ANI results^2^ (Supplementary Table 1). To date, infections of *C. thiooxydans* have not yet been reported. This current study describes the bacterium *C. thiooxydans* ZDHYF418, which has been isolated from a patient’s urine specimen.

The IMP-type metallo beta-lactamase (MBL) was first reported in Japan in 1991 when the *bla*_*IMP–*__1_ was identified in a *Pseudomonas aeruginosa* isolate (Watanabe et al., 1991). The IMP family spread to various areas including Europe (Riccio et al., 2000), China (Hawkey et al., 2001), Australia (Peleg et al., 2004), and the United States (Hanson et al., 2006). The *bla*_*IMP–*__8_ was first identified from *Klebsiella pneumoniae* in Taiwan in 2001, it is a variant of *bla*_*IMP–*__2_ with four nucleotide differences, which resulting in two amino acid differences (Yan et al., 2001). Subsequently, it were also found in *Pseudomonas mendocina* in Portugal (Santos et al., 2010), *Enterobacter cloacae* in Argentina (Gomez et al., 2011), *Klebsiella oxytoca* in Spain (Vergara-Lopez et al., 2013), and *Klebsiella pneumoniae* in Tunisia (Chouchani et al., 2013). To date, *bla*_*IMP*_ genes have not been identified in *C. thiooxydans*. In this study, we set out to describe the isolation of an IMP-8-producing *C. thiooxydans* from a patient with a urinary tract infection in China. This is the first time that a carbapenem resistance (CR) gene has been found in *C. thiooxydans*. Therefore, it is necessary to carry out further study on this strain, the genomic and phenotypic characteristics.

## Materials and Methods

### Species Identification and Antimicrobial Susceptibility Testing

Species identification was performed using MALDI-TOF/MS (Bruker Daltonik GmbH, Bremen, Germany), as well as average nucleotide identity (ANI). Carbapenemase-encoding genes were detected using PCR. Supplementary Table 2 contains data regarding specific experimental conditions and primer sequence information. Antimicrobial Susceptibility Testing (AST) was carried out using agar dilution, and results were interpreted according to CLSI 2020 standards for other non-enterobacterales bacteria (Clinical and Laboratory Standards Institute, 2020). The strains *Escherichia coli* ATCC 25922 and *P. aeruginosa* ATCC 27853 were used as controls.

### Whole Genome Sequencing and Bioinformatics Analysis

Genomic DNA was extracted using a Genomic DNA Isolation Kit (QIAGEN, Hilden, Germany) and sequenced using Illumina Novaseq (Illumina, Inc., CA, United States) and Oxford Nanopore PromethION (Oxford Nanopore Technologies, Oxford, United Kingdom). Draft genomes were obtained using SPAdes version 3.9.1^3^ and annotated by the NCBI Prokaryotic Genome Annotation Pipeline (PGAP)^4^ and RAST version 2.0^5^ (Aziz et al., 2008). Acquired antibiotic resistance genes were identified using ResFinder version 3.2^6^ and CARD^7^. Comparison of the genetic structures carrying *bla*_*IMP–*__8_ gene were performed through Easyfig version 2.2.3 (Sullivan et al., 2011).

### Phylogenetic Analysis

In order to investigate the phylogenetic relationships between *C. thiooxydans* ZDHYF418 and additional *C. thiooxydans* strains, we downloaded all 21 *C. thiooxydans* publicly available genomes in the NCBI Genome database^8^ as of October 2020 (Supplementary Table 1). Next, we used KSNP 3.0 (Gardner et al., 2015) to create a SNP-based phylogenetic tree through the use of *C. thiooxydans* CNB (accession number: CP001220.2) as reference strain. The phylogenetic tree was subsequently visualized and modified using iTOL version 5^9^.

## Results and Discussion

### Case Description

We isolated a novel strain of *C. thiooxydans*, designated as ZDHYF418, from the mid-section urine specimen of a 60-year-old female patient that was admitted to a public hospital in Zhengzhou, China in 2019. The patient was admitted to the hospital for treatment of left kidney stones. During hospitalization, the patient was intermittently irritable, unconscious, and went into septic shock. Additionally, the patient experienced abdominal distension, nausea, and vomiting. The blood culture results indicated the presence of an *E. coli* infection. The patient was administered imipenem-cilastatin 1g ivgtt Q8H for 1 month to fight the infection. The doctor plans to perform transurethral ureteroscopy with lithotripsy when her condition became stable. *C. thiooxydans* was detected in the patient’s urine culture the day before the surgery. The patient’s stones suddenly recurred and the pain could not be relieved, so the doctor did not postpone the operation. The patient was treated for a week until her condition stabilized and she was discharged from the hospital.

### Strain Identification of ZDHYF418

Strain ZDHYF418 was named *C. thiooxydans* after the observation of ANIs analysis based on BLAST. In fact, the genomic sequences of ZDHYF418 are 96.830% identical by ANI to the genome of *C. thiooxydans*, with 86.2% coverage of the genome. This result reveals that a phylogenetic affiliation of strain ZDHYF418 belongs to the species *C. thiooxydans.*

### Resistome of *C. thiooxydans* ZDHYF418

According to CLSI 2020 standards for other non-enterobacterales bacteria, *in vitro* susceptibility tests results indicated that ZDHYF418 is a multi-drug resistant strain (Table 1). Antibiogram assays indicated that ZDHYF418 is resistant to most of the antibiotics tested in this study. ZDHYF418 was found to be resistant to ceftazidime, cefepime, levofloxacin, ciprofloxacin, amikacin, gentamicin, and aztreonam; intermediate to ceftriaxone, piperacillin/tazobactam, and meropenem; susceptible to imipenem, trimethoprim/sulfamethoxazole, and chloramphenicol. According to the analysis of ResFinder and CARD, *bla*_*IMP–*__8_ and *aac(6′)-Ib-cr* are all resistance genes contained in the sequence of strain ZDHYF418. In addition, gyrA, parC, and a novel class D beta-lactamase gene *bla*_*OXA*_ were also found in the annotation results. The *bla*_*IMP–*__8_ and *bla*_*OXA*_ genes confer resistance to β-lactam antibiotics, β-lactamase production is the most common resistance mechanism. The *aac(6′)-Ib-cr* gene mainly modifies the amino group of aminoglycosides to inactivate aminoglycoside antibiotics, thereby conferring resistance to aminoglycosides. In addition, the resistance to fluoroquinolones is mainly caused by mutations in the coding regions of the gyrase subunit (gyrA) and DNA topoisomerase IV (parC) (Hawkey and Jones, 2009). Usually, the *bla*_*IMP*_ gene confers resistance to beta-lactam antibiotics, except monobactams. However, our data indicates that ZDHYF418 is resistant to aztreonam. According to the annotation results of ZDHYF418, a series of efflux pumps such as efflux RND transporter, multidrug effflux MFS transporter, MacB family efflux pump can be found (Cattoir, 2004; Braz et al., 2016). The efflux pump system is one of the most essential resistance mechanisms. We do not know the expression of the pumps exactly, but we suppose that the resistance of ZDHYF418 to aztreonam might be similar to that in pseudomonas.

**TABLE 1:** Minimum inhibitory concentration (MIC) values of antimicrobials for *Comamonas thiooxydans* ZDHYF418.

Antibiotics	MICs (mg/L)	Interpretation
**β -lactam**		
Ceftazidime	>64	R
Ceftriaxone	16	I
Cefepime	>64	R
Piperacillin/Tazobactam	32	I
Imipenem	2	S
Meropenem	8	I
Aztreonam	>64	R
**Fluoroquinolone**		
Levofloxacin	32	R
Ciprofloxacin	32	R
**Aminoglycoside**		
Amikacin	128	R
Gentamicin	>16	R
Sulfonamide		
Trimethoprim/sulfamethoxazole	0.25	S
**Other/**		
Chloramphenicol	8	S

*R, resistant; S, susceptible; I, intermediate.*

### Genetic Structure of *bla*_*IMP–*__8_ Gene in *C. thiooxydans* ZDHYF418

**FIGURE 1:**
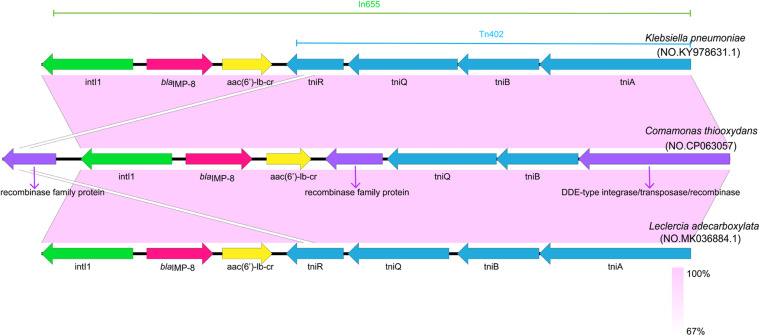
Comparison of the genetic structure surrounding *bla*_*IMP–*__8_ in *Comamonas thiooxydans* ZDHYF418 and those observed in other *bla*_*IMP–*__8_ positive bacteria. Open reading frames (ORFs) are indicate as arrows that show the orientation of coding sequence with the gene name. Regions with a high degree of homology are indicated by light purple shading.

We report here the genomic sequence of this strain contains 5,273,527 bp with a GC content of 61.4%. The genetic structure of the *bla*_*IMP–*__8_ gene in ZDHYF418 and the sequences most similar to ZDHYF418 by BLAST are shown in Figure 1. The genetic structure around *bla*_*IMP–*__8_ in *C. thiooxydans* ZDHYF418 has a percent identity of 99.96 and 99.94% to the p447-IMP in *K. pneumoniae* (accession number: KY978631) (Zhan et al., 2018) and p16005813B in *Leclercia adecarboxylata*, respectively (accession number: MK036884) (Yin et al., 2019). The genetic structure of *bla*_*IMP–*__8_ gene in *C. thiooxydans* ZDHYF418 includes *DDE-type integrase/transposase/recombinase–tniB–tniQ-recombinase family protein-aac(6′)-Ib-cr-bla_*IMP–*__8_-intI1*. p447-IMP and p16005813B both contain the In*655* integron, which is an ancestral Tn*402*-associated integron. The differences among these sequences are that in ZDHYF418, the tniR module may be missing and replaced by a recombinase family protein. It has a 100% identity to MULTISPECIES: recombinase family protein (accession number: WP_003155741.1), it belongs to the serine recombinase (SR) family that can mediate site-specific recombination (Stark, 2014). The tniA module may be missing and is replaced by a DDE-type integrase/transposase/recombinase. It has a 100% identity to MULTISPECIES: DDE-type integrase/transposase/recombinase (accession number:WP_088244042.1). Both of the recombinase family protein and DDE-type integrase/transposase/recombinase are non-redundant protein sequences.

### Analysis of Phylogenetic Relationships

**FIGURE 2:**
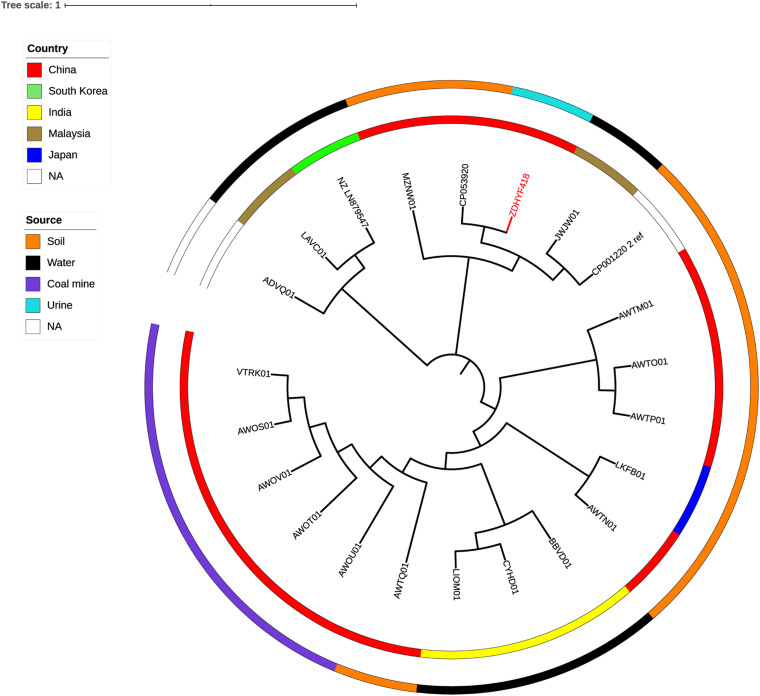
A phylogenetic tree showing *C. thiooxydans* strain ZDHYF418 along with all additional *C. thiooxydans* genomes are publicly available in the NCBI Genome database. The *C. thiooxydans* ZDHYF418 strain is indicated in red. The two circles around the phylogenetic tree indicate country of origin (inner circle) and the source (outer circle) of these strains. NA, not applicable.

The phylogenetic relationship of *C. thiooxydans* ZDHYF418 to the 21 *C. thiooxydans* are depicted in Figure 2 and Supplementary Tables 1, 3. According to Figure 2 and Supplementary Table 3, the closest relative of ZDHYF418 is *C. thiooxydans* QYY (accession number: CP053920.1). There are 330 SNP differences between ZDHYF418 and *C. thiooxydans* QYY. Strain QYY was isolated from activated sludge in Jilin province in 2015. On the other hand, *C. thiooxydans* has been mainly isolated from the environment, especially soil (Figure 2 and Supplementary Tables 1, 3). AWTM01, AWTP01, and AWTO01 come from the same country, as well as the same source, and their relationships are the closest. Furthermore, AWOT01, AWOU01, AWOV01, AWOS01, and VTRK01 come from the same country and the same source, and they are closely related. LIOM01 and CYHD01 come from the same country and source, and their relationship is the closest.

### Clinical Perspectives

In this study, we describe a *C. thiooxydans* strain from the urine of a patient with urinary tract infection caused by *E. coli* and *C. thiooxydans*. The patient ultimately developed septic shock. According to a previous case report of septic shock caused by bloodstream infection (Grumaz et al., 2016), we speculate that septic shock in our patient may also be due to a bloodstream infection. Additionally, in our case, the patient was fitted with a urinary catheter during hospitalization, and therefore, the bloodstream infection may be caused by a retrograde urethral infection. However, we were unable to collect a specific catheter for testing. Therefore, it is impossible to trace the source of infection. Our experience of this case highlights the need for increased awareness with regards to hospital-acquired infections caused by *C. thiooxydans.*

## Conclusion

In summary, we first identified a *bla*_*IMP–*__8_-positive *C. thiooxydans* strain from a human urine sample. The isolation of *C. thiooxydans* from humans is very rare, and the strain we identified was clearly resistant to aztreonam, fluoroquinolones and aminoglycosides, and intermediate to meropenem. The increased resistance of bacteria to antibiotics is now starting to appear in less common bacteria, such as *C. thiooxydans*. This finding prompts us to standardize clinical medication and pay more attention to bacterial resistance monitoring of this species.

## Data Availability Statement

The datasets generated for this study can be found in NCBI under BioProject PRJNA623107, https://www.ncbi.nlm.nih.gov/bioproject/?term=623107.

## Ethics Statement

Written informed consent was obtained from the participant of this study. Ethical permission was granted by the Research Ethics Committee of The First Affiliated Hospital, College of Medicine, Zhejiang University, reference number 2018#752.

## Author Contributions

XG and JG conceived and designed the experiments. QW, SL, and PW collected clinical samples and performed the experiments. HX, XH, and LG analyzed the data. QW wrote the manuscript. All authors contributed to the article and approved the submitted version.

## Conflict of Interest

The authors declare that the research was conducted in the absence of any commercial or financial relationships that could be construed as a potential conflict of interest.

## Footnotes

1 http://www.bacterio.net/comamonas.html

2 https://www.ncbi.nlm.nih.gov/genome/browse/#!/prokaryotes/36735/

3 http://cab.spbu.ru/software/spades/

4 https://www.ncbi.nlm.nih.gov/genome/annotation_prok/

5 https://rast.nmpdr.org/

6 https://cge.cbs.dtu.dk/services/ResFinder/

7 https://card.mcmaster.ca/

8 https://www.ncbi.nlm.nih.gov/genome/genomes/

9 https://itol.embl.de/

## References

[B1] Aziz R. K. Bartels D. Best A. A. DeJongh M. Disz T. Edwards R. A. (2008). The RAST server: rapid annotations using subsystems technology. *BMC Genomics* 9:75. 10.1186/1471-2164-9-75 18261238 PMC2265698

[B2] Braz V. S. Furlan J. P. Fernandes A. F. Stehling E. G. (2016). Mutations in NalC induce MexAB-OprM overexpression resulting in high level of aztreonam resistance in environmental isolates of *Pseudomonas aeruginosa*. *FEMS Microbiol. Lett.* 363:fnw166. 10.1093/femsle/fnw166 27412168

[B3] Cattoir V. (2004). [Efflux-mediated antibiotics resistance in bacteria]. *Pathol. Biol. (Paris)* 52 607–616. 10.1016/j.patbio.2004.09.001 15596311

[B4] Chen Y. L. Wang C. H. Yang F. C. Ismail W. Wang P. H. Shih C. J. (2016). Identification of *Comamonas testosteroni* as an androgen degrader in sewage. *Sci. Rep.* 6:35386. 10.1038/srep35386 27734937 PMC5062160

[B5] Chouchani C. Marrakchi R. Henriques I. Correia A. (2013). Occurrence of IMP-8, IMP-10, and IMP-13 metallo-beta-lactamases located on class 1 integrons and other extended-spectrum beta-lactamases in bacterial isolates from Tunisian rivers. *Scand. J. Infect. Dis.* 45 95–103. 10.3109/00365548.2012.717712 22992193

[B6] Clinical and Laboratory Standards Institute (2020). *Performance Standards for Antimicrobial Susceptibility Testing, CLSI supplement M100*, 30th Edn. Wayne, PA: Clinical and Laboratory Standards Institute.

[B7] Gardner S. N. Slezak T. Hall B. G. (2015). kSNP3.0: SNP detection and phylogenetic analysis of genomes without genome alignment or reference genome. *Bioinformatics* 31 2877–2878. 10.1093/bioinformatics/btv271 25913206

[B8] Gomez S. Rapoport M. Togneri A. Viegas-Caetano J. Faccone D. Corso A. (2011). Emergence of metallo-beta-lactamases in Enterobacteriaceae from Argentina. *Diagn. Microbiol. Infect. Dis.* 69 94–97. 10.1016/j.diagmicrobio.2010.08.025 21146720

[B9] Grumaz S. Stevens P. Grumaz C. Decker S. O. Weigand M. A. Hofer S. (2016). Next-generation sequencing diagnostics of bacteremia in septic patients. *Genome Med.* 8:73. 10.1186/s13073-016-0326-8 27368373 PMC4930583

[B10] Hanson N. D. Hossain A. Buck L. Moland E. S. Thomson K. S. (2006). First occurrence of a *Pseudomonas aeruginosa* isolate in the United States producing an IMP metallo-beta-lactamase, IMP-18. *Antimicrob. Agents Chemother.* 50 2272–2273. 10.1128/aac.01440-05 16723605 PMC1479107

[B11] Hatayama K. (2014). *Comamonas humi* sp. nov., isolated from soil. *Int. J. Syst. Evol. Microbiol.* 64(Pt 12) 3976–3982. 10.1099/ijs.0.067439-0 25212224

[B12] Hawkey P. M. Jones A. M. (2009). The changing epidemiology of resistance. *J. Antimicrob. Chemother.* 64(Suppl. 1) i3–i10. 10.1093/jac/dkp256 19675017

[B13] Hawkey P. M. Xiong J. Ye H. Li H. M’Zali F. H. (2001). Occurrence of a new metallo-beta-lactamase IMP-4 carried on a conjugative plasmid in *Citrobacter youngae* from the people’s republic of China. *FEMS Microbiol. Lett.* 194 53–57. 10.1111/j.1574-6968.2001.tb09445.x 11150665

[B14] Kämpfer P. Busse H. J. Baars S. Wilharm G. Glaeser S. P. (2018). *Comamonas aquatilis* sp. nov., isolated from a garden pond. *Int J Syst Evol Microbiol* 68 1210–1214. 10.1099/ijsem.0.002652 29473819

[B15] Narayan K. D. Badhai J. Whitman W. B. Das S. K. (2016). Draft genome sequence of *Comamonas thiooxydans* strain S23T (DSM 17888T), a thiosulfate-oxidizing bacterium isolated from a sulfur spring in India. *Genome Announc.* 4 e834–e816. 10.1128/genomeA.00834-16 27516520 PMC4982299

[B16] Pandey S. K. Narayan K. D. Bandyopadhyay S. Nayak K. C. Das S. K. (2009). Thiosulfate oxidation by *Comamonas* sp. S23 isolated from a sulfur spring. *Current Microbiology* 58 516–521. 10.1007/s00284-009-9357-3 19189181

[B17] Peleg A. Y. Franklin C. Bell J. Spelman D. W. (2004). Emergence of IMP-4 metallo-beta-lactamase in a clinical isolate from Australia. *J. Antimicrob. Chemother.* 54 699–700. 10.1093/jac/dkh398 15282242

[B18] Riccio M. L. Franceschini N. Boschi L. Caravelli B. Cornaglia G. Fontana R. (2000). Characterization of the metallo-beta-lactamase determinant of *Acinetobacter baumannii* AC-54/97 reveals the existence of bla(IMP) allelic variants carried by gene cassettes of different phylogeny. *Antimicrob. Agents Chemother.* 44 1229–1235. 10.1128/aac.44.5.1229-1235.2000 10770756 PMC89849

[B19] Santos C. Caetano T. Ferreira S. Mendo S. (2010). First description of bla IMP-8 in a *Pseudomonas mendocina* isolated at the Hospital Infante D. Pedro, Aveiro, Portugal. *Res. Microbiol.* 161 305–307. 10.1016/j.resmic.2010.03.004 20381610

[B20] Stark W. M. (2014). The serine recombinases. *Microbiol. Spectr.* 2:6. 10.1128/microbiolspec.MDNA3-0046-2014 26104451

[B21] Subhash Y. Bang J. J. You T. H. Lee S. S. (2016). Description of *Comamonas sediminis* sp. nov., isolated from lagoon sediments. *Int. J. Syst. Evol. Microbiol.* 66 2735–2739. 10.1099/ijsem.0.001115 27117992

[B22] Sullivan M. J. Petty N. K. Beatson S. A. (2011). Easyfig: a genome comparison visualizer. *Bioinformatics* 27 1009–1010. 10.1093/bioinformatics/btr039 21278367 PMC3065679

[B23] Tsui T. L. Tsao S. M. Liu K. S. Chen T. Y. Wang Y. L. Teng Y. H. (2011). *Comamonas testosteroni* infection in Taiwan: reported two cases and literature review. *J. Microbiol. Immunol. Infect.* 44 67–71. 10.1016/j.jmii.2011.01.013 21531356

[B24] Vergara-Lopez S. Dominguez M. C. Conejo M. C. Pascual A. Rodriguez-Bano J. (2013). Wastewater drainage system as an occult reservoir in a protracted clonal outbreak due to metallo-beta-lactamase-producing *Klebsiella oxytoca*. *Clin. Microbiol. Infect.* 19 E490–E498. 10.1111/1469-0691.12288 23829434

[B25] Watanabe M. Iyobe S. Inoue M. Mitsuhashi S. (1991). Transferable imipenem resistance in *Pseudomonas aeruginosa*. *Antimicrob. Agents Chemother.* 35 147–151. 10.1128/aac.35.1.147 1901695 PMC244956

[B26] Willems A. De Vos P. (2006). “Comamonas,” in *The Prokaryotes*, eds Dworkin M. Falkow S. Rosenberg E. Schleifer K.-H. Stackebrandt E. (New York, NY: Springer), 723–736.

[B27] Yan J. J. Ko W. C. Wu J. J. (2001). Identification of a plasmid encoding SHV-12, TEM-1, and a variant of IMP-2 metallo-beta-lactamase, IMP-8, from a clinical isolate of *Klebsiella pneumoniae*. *Antimicrob. Agents Chemother.* 45 2368–2371. 10.1128/AAC.45.8.2368-2371.2001 11451699 PMC90656

[B28] Yin Z. Hu L. Cheng Q. Jiang X. Xu Y. Yang W. (2019). First report of coexistence of three different MDR plasmids, and that of occurrence of IMP-encoding plasmid in *Leclercia adecarboxylata*. *Front. Microbiol.* 10:2468. 10.3389/fmicb.2019.02468 31749779 PMC6848029

[B29] Zhan Z. Hu L. Jiang X. Zeng L. Feng J. Wu W. (2018). Plasmid and chromosomal integration of four novel blaIMP-carrying transposons from *Pseudomonas aeruginosa*, *Klebsiella pneumoniae* and an *Enterobacter* sp. *J. Antimicrob. Chemother.* 73 3005–3015. 10.1093/jac/dky288 30351436

[B30] Zhou Y. H. Ma H. X. Dong Z. Y. Shen M. H. (2018). *Comamonas kerstersii* bacteremia in a patient with acute perforated appendicitis: a rare case report. *Medicine (Baltimore)* 97:e9296. 10.1097/md.0000000000009296 29595695 PMC5895375

