# Supplementary material for: Emergence of IMP-8-Producing Comamonas thiooxydans Causing Urinary Tract Infection in China
Source: Front Microbiol. 2021 Mar 15;12:585716. doi: 10.3389/fmicb.2021.585716 (PMC8005532; doi:10.3389/fmicb.2021.585716)
Supplement: Supplementary file 2 [file Table_2.DOCX]

Table S2. PCR primers and conditions

| PCR Primer | sequence |
| --- | --- |
| NDM-F | ATGGAATTGCCCAATATTATGCAC |
| NDM-R | TCAGCGCAGCTTGTCGGC |
| KPC-F | ATGTCACTGTATCGCCGTC |
| KPC-R | TTACTGCCCGTTGACGCC |
| IMP-F | GTTTATGTTCATACWTCG |
| IMP-R | GGTTTAAYAAAACAACCAC |
| VIM-F | TTTGGTCGCATATCGCAACG |
| VIM-R | CCATTCAGCCAGATCGGCAT |
| OXA-48-F | ATGAAAAAATTTATACTTCC |
| OXA-48-R | TTAAATGATTCCAAGATTTTC |
| OXA-1-F | ACACAATACATATCAACTTCGC |
| OXA-1-R | GTGTGTTTAGAATGGTGATC |
| OXA-10-F | CCACCAAGAAGGTGCCATGA |
| OXA-10-R | GCGACCTTGAGCGACTTGTT |
| TEM-F | CATTTCCGTGTCGCCCTTATTC |
| TEM-R | CGTTCATCCATAGTTGCCTGAC |
| MCR1-F | CGCCATATGTGCAGCATACTTCTGTGTGG |
| MCR1-R | CCGCTCGAGGGTGCGGTCTTTGACTTTG |
| MCR2-F | GCGATGGCGGTCTATCCTGTATCGG |
| MCR2-R | GGCTGACACCCCATGTCATCGCACG |

PCR reaction system

| ingredient | Volume (μL) |
| --- | --- |
| 2× Taq Master Mix enzyme | 25 |
| primer-F | 1 |
| primer-R | 1 |
| DNA | 2 |
| ddH_2_O | 21 |
| Total | 50 |

PCR reaction conditions

| Temperature | Time | Cycle |
| --- | --- | --- |
| 94℃ | 5min | 1 |
| 94℃ | 1min | 30 |
| 55℃ | 30s | 30 |
| 72℃ | 1min | 30 |
| 72℃ | 7min | 1 |
| 4℃ | 1h | 0 |

IMP gene PCR reaction conditions

| Temperature | Time | Cycle |
| --- | --- | --- |
| 94℃ | 5min | 1 |
| 94℃ | 1min | 30 |
| 42℃ | 30s | 30 |
| 72℃ | 1min | 30 |
| 72℃ | 7min | 1 |
| 4℃ | 1h | 0 |
